# First person – Kate Quigley

**DOI:** 10.1242/bio.050427

**Published:** 2020-01-23

## Abstract

First Person is a series of interviews with the first authors of a selection of papers published in Biology Open, helping early-career researchers promote themselves alongside their papers. Kate Quigley is first author on ‘Assessing the role of historical temperature regime and algal symbionts on the heat tolerance of coral juveniles’, published in BIO. Kate is a postdoc in the lab of Madeleine van Oppen and Line Bay at Australian Institute of Marine Science, Australia, investigating the genomic mechanisms associated with population connectivity, thermal tolerance and adaptation, and resiliency of coral reef organisms.


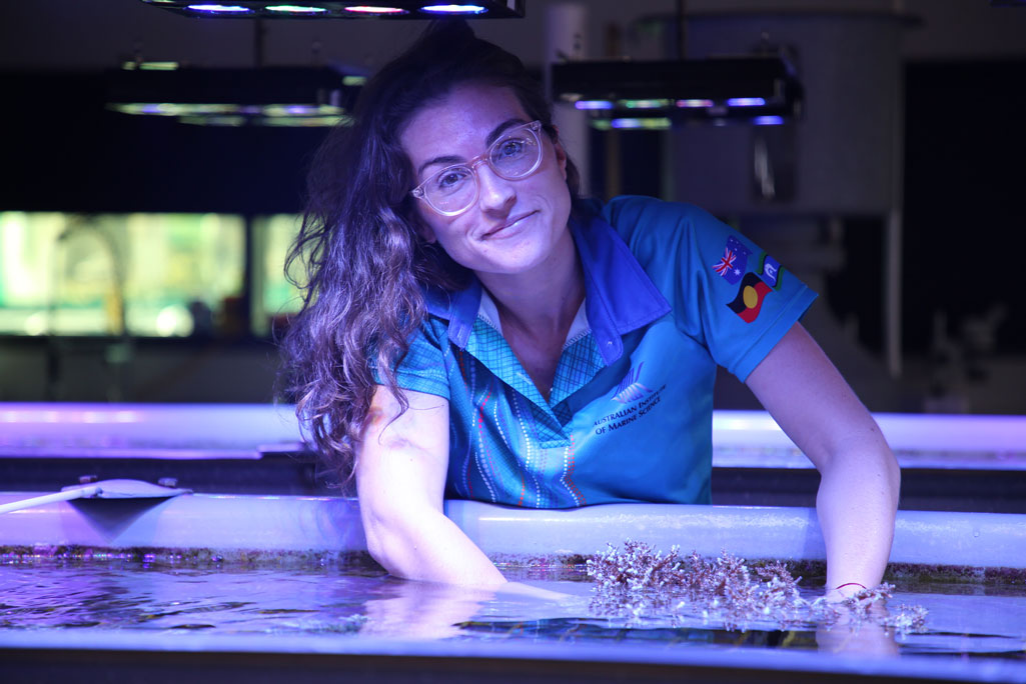


**Kate Quigley holding an adult coral at the National Sea Simulator (Seasim) at the Australian Institute of Marine Science (AIMS).**

**What is your scientific background and the general focus of your lab?**

I am an integrative molecular ecologist with a focus on evolutionary genomics of corals from a host–symbiont perspective. My research interests include investigating the genomic mechanisms associated with population connectivity, thermal tolerance and adaptation and resiliency of coral reef organisms.

I currently hold a Postdoctoral Research Fellowship at the Australian Institute of Marine Science (AIMS) investigating the genomic basis of environmental stress tolerance and resilience in corals. In this position, I use population genomic and quantitative genetic theory in conjunction with field, experimental and modelling methods to develop research frameworks for testing the feasibility of large-scale restoration interventions. Previously during my PhD, I incorporated methods related to symbiosis and coral ecology, reproductive biology and physiology to determine the impacts of symbiosis on the early life-history stages of corals.

I am part of the adaptation and resilience of coral reefs team at AIMS, led by Dr Line Bay and Prof. Madeleine van Oppen. The general focus of the lab is to integrate physiological, genetic and genomic data to understand how corals interact with their environment. In particular, we focus on rates and mechanisms of physiological acclimatisation, and the potential for genetic adaptation in response to climate and ocean change.

“We are looking for new ways to help corals resist higher temperatures and prevent them from bleaching.”

**Figure:**
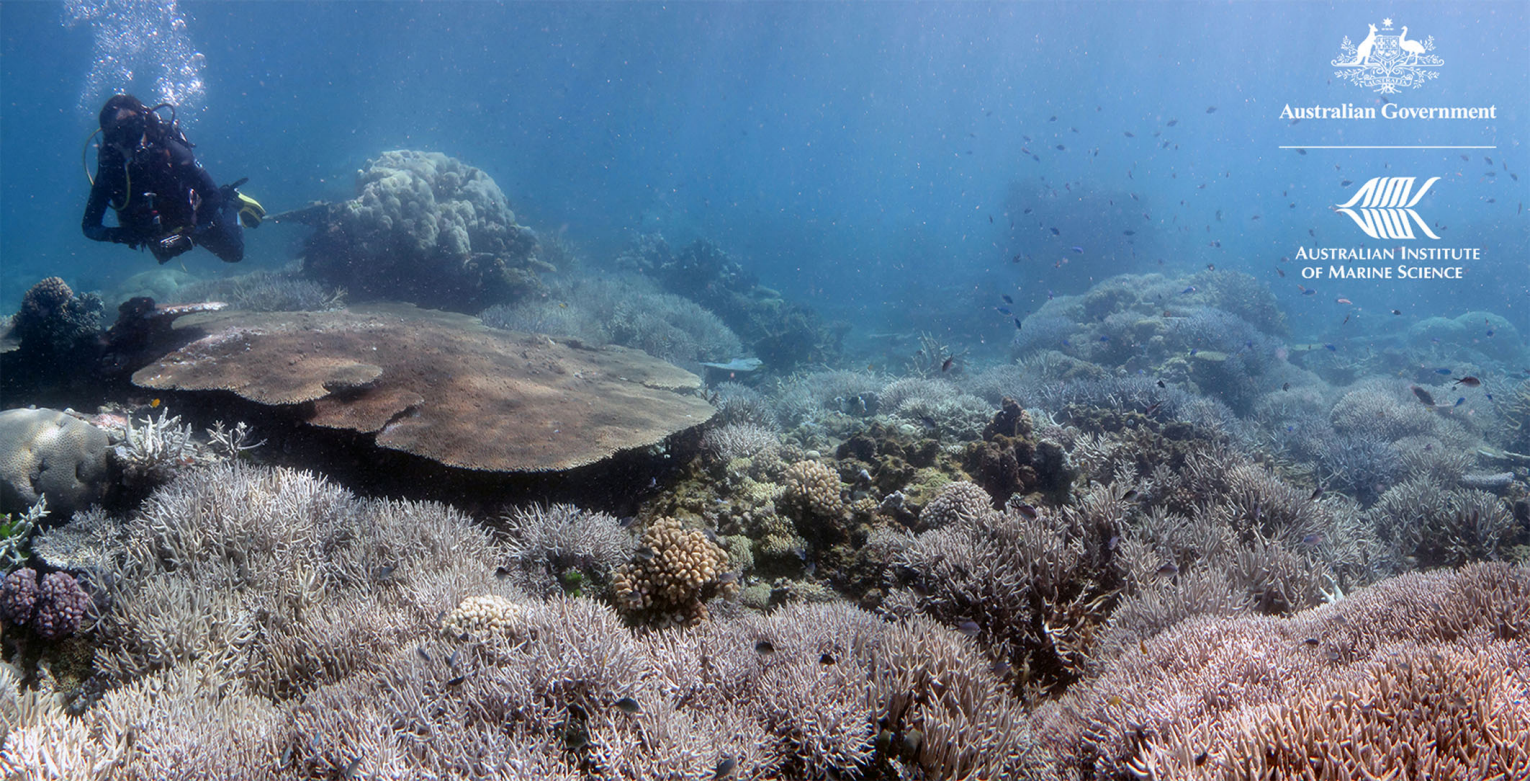
**One of the reef locations where breeding parental corals were collected in the far north of the Great Barrier Reef before coral spawning.**

**How would you explain the main findings of your paper to non-scientific family and friends?**

Corals live in a symbiotic relationship with many microbes, including tiny photosynthetic algae that provide the coral with most of their energy. When temperatures become warm this relationship breaks down, causing the algae to be lost from the coral animal and turning it white and at times leading to the coral's death. This is known as coral bleaching.

We are looking for new ways to help corals resist higher temperatures and prevent them from bleaching.

**What are the potential implications of these results for your field of research?**

These results demonstrate that it is possible to significantly increase the bleaching tolerance and survival of coral juveniles when exposed to high seawater temperatures using currently available methods like selective breeding and directed symbiosis establishment.

We hope that these results can be applied as a potential restoration method not only on the Great Barrier Reef in Australia (after due diligence), but hopefully other reefs worldwide that are suffering from coral loss.

**What has surprised you the most while conducting your research?**

I think the greatest surprise was the large effect size of the symbiont treatment. Although it is quite established that the symbiotic partner is one of the key drivers in bleaching resistance and susceptibility, it was great news to see how substantial the effect of the symbionts were. This is positive news for developing novel interventions based on symbiont additions.

It was also encouraging that we did not detect large trade-offs between increased heat tolerance and decreased growth in coral juveniles. Other studies have found such trade-offs between reproductive potential and thermal acclimation, so we were worried that any benefits caused by selective breeding would be jeopardised by reductions in growth. Thankfully, we did not observe this with these particular populations tested.

“[…] it was great news to see how substantial the effect of the symbionts were.”

**What, in your opinion, are some of the greatest achievements in your field and how has this influenced your research?**

In my opinion, some of the greatest achievements in my field were the discovery of the two symbiont-derived mechanisms that corals use to acclimate quickly to changes in seawater temperature. These are called ‘shuffling’ and ‘switching’ and involve either changes in relative abundance of symbionts inside of corals to a more heat-tolerant taxa (shuffling), or the novel uptake of potentially heat-tolerant symbionts from the environment (switching). The discovery of these mechanisms opened up new fields of research to characterise and document coral bleaching worldwide and to understand the mechanisms influencing bleaching responses.

It has also led to the search for other novel symbiont-driven acclimatory and adaptive mechanisms that could further help corals cope with warming oceans, and shed light on why some corals are more susceptible to bleaching and death whilst others are not. Almost two decades of studying these mechanisms suggests that warming rate, its severity, and the time interval between bleaching influences how the coral–symbiont partnership responds and ultimately how corals may fare in the future.

**What changes do you think could improve the professional lives of early-career scientists?**

Early-career scientists could benefit greatly from mentoring on how to establish and maintain successful links and collaborations between academia and industry. It is well known that the vast majority of graduating PhDs will not go on to tenured-track positions at universities. Therefore, training on how to attain ‘alternative’ (is it ‘alternative’ if a majority undertake them?) career paths should be part of early-career scientist training.

Additional and specific funding for early-career scientists would also help to establish more independent careers earlier whilst still under the mentorship of PIs. Specifically, this funding should recognise other factors beyond publication numbers as a metric for success, for example, cross-disciplinary research or outreach in the community.

**What's next for you?**

My plan at AIMS is to continue to explore the temperature tolerance of corals sourced from many other locations around the Great Barrier Reef. These expeditions will hopefully allow us to find reefs, populations and individuals that are particularly hardy and that will act as breeding stock for the production of coral offspring able to withstand high heat. We have thus far surveyed and spawned corals from seven additional reefs, encompassing many degrees of latitude. The search for hardy and resilient corals continues, but we have already seen some very promising results!

## References

[BIO050427C1] Quigley K. M., Randall C. J., van Oppen M. J. H. and Bay L. K. (2020). Assessing the role of historical temperature regime and algal symbionts on the heat tolerance of coral juveniles. *Biology Open* 9, 047316 10.1242/bio.047316 PMC6994947 31915210

